# Cohort Profile Update: 2004 Pelotas (Brazil) Birth Cohort Study follow-up during adolescent years

**DOI:** 10.1093/ije/dyad156

**Published:** 2023-11-09

**Authors:** Luciana Tovo-Rodrigues, Iná S Santos, Isabel Oliveira Bierhals, Bianca Del-Ponte, Joseph Murray, Renata Bielemann, Tiago N Munhoz, Inácio Crochemore-Silva, Isabel O de Oliveira, Fernando C Barros, Aluísio J D Barros, Alicia Matijasevich

**Affiliations:** Postgraduate Program in Epidemiology, Federal University of Pelotas, Pelotas, RS, Brazil; Human Development and Violence Research Centre, Federal University of Pelotas, Pelotas, RS, Brazil; Postgraduate Program in Epidemiology, Federal University of Pelotas, Pelotas, RS, Brazil; Postgraduate Program in Epidemiology, Federal University of Pelotas, Pelotas, RS, Brazil; Postgraduate Program in Epidemiology, Federal University of Pelotas, Pelotas, RS, Brazil; Postgraduate Program in Epidemiology, Federal University of Pelotas, Pelotas, RS, Brazil; Human Development and Violence Research Centre, Federal University of Pelotas, Pelotas, RS, Brazil; Postgraduate Program in Nutrition and Foods, Federal University of Pelotas, Pelotas, RS, Brazil; Postgraduate Program in Epidemiology, Federal University of Pelotas, Pelotas, RS, Brazil; School of Psychology, Federal University of Pelotas, Pelotas, RS, Brazil; Postgraduate Program in Epidemiology, Federal University of Pelotas, Pelotas, RS, Brazil; Postgraduate Program in Physical Education, Federal University of Pelotas, Pelotas, RS, Brazil; Postgraduate Program in Epidemiology, Federal University of Pelotas, Pelotas, RS, Brazil; Postgraduate Program in Health in the Life Cycle, Universidade Catolica de Pelotas, Pelotas, RS, Brazil; Postgraduate Program in Epidemiology, Federal University of Pelotas, Pelotas, RS, Brazil; Postgraduate Program in Epidemiology, Federal University of Pelotas, Pelotas, RS, Brazil; Departamento de Medicina Preventiva, Faculdade de Medicina FMUSP, Universidade de São Paulo, São Paulo, SP, Brasil

**Keywords:** Adolescents, cohort study, mental health, non-communicable diseases, clinical conditions, human capital, violence, COVID-19 pandemic impact, low- and middle-income countries (LMIC)

Key Features

- The 2004 Pelotas Birth Cohort is a population-based study in the city of Pelotas, Brazil. The 4231 participants included at birth were assessed at 3, 12, 24 and 48 months, and at 6, 11, 15 and 17 years of age.
- Here we present information on the 11-year and 15-year follow-ups and the COVID-19 impact study at the 17-year follow-up.
- Data collected included: measures of mental health; risk factors for non-communicable diseases; clinical conditions; human capital; violence; and the impact of the COVID-19 pandemic. We assessed maternal cognition and mental health.
- We assessed 86.6% (*N* = 3566) of the baseline cohort at the 11-year follow-up. Due to the COVID-19 pandemic, the 15-year follow-up was halted when we had assessed 1949 individuals (48.5% of the cohort). Those seen at age 15 years were reassessed at the 17-year follow-up with the COVID-19 impact study (*n* = 1826).
- For collaboration requests, please refer to our website [https://www.epidemio-ufpel.org.br] or contact the corresponding author.

## The original cohort

The 2004 Pelotas Birth Cohort is a large population-based study of all live babies born from 1 January to 31 December 2004 to women living in the urban area of the city of Pelotas, Brazil. All hospitals with maternity wards were visited daily, and all live births (*N * =  4263) were eligible for enrolment in the cohort. A total of 4231 newborns, 99.2% of all births in the city that year, were included in the study.^1^ The main objectives of the original study were to investigate the impact of early life exposures on health outcomes, and to study social inequities in health conditions.^1^ After a first assessment at the time of birth, all cohort participants were assessed at ages 3, 12, 24, and 48 months and at age 6 years, with follow-up rates of 95.7%, 94.3%, 93.5%, 92.0% and 90.2%, respectively.^2^ A wide range of measures have already been taken, including assessment of general and mental health, body composition, development and cognitive ability of the participants, family environment and maternal general and health. More recently, participants were assessed at ages 11, 15 and 17 years.

## What is the reason for the new focus and new data collection?

In this update we describe the assessments at 11 years (in 2015), and 15 years (in 2019–20) and a reassessment of the participants seen at the 15-year follow-up, during the COVID-19 pandemic (in 2021), when they turned 17 years old (COVID-19 impact study). As adolescence is a critical period for mental and physical health, additional questions were included in the subsequent waves of data collection. These follow-up visits had a special focus on various aspects of mental health and their potential determinants. Additionally, at age 15 years and during the COVID-19 impact study, we collected hair samples to measure cortisol concentration, which has been used as a marker of chronic stress.

By March 2020, when the COVID-19 pandemic hit Brazil, all research studies in the country, especially those involving face-to-face fieldwork, were interrupted. Therefore, the 15-year follow-up of the 2004 Pelotas Birth Cohort, which had started in November 2019, stopped midway, having assessed only half the participants. Even though children and adolescents are at a lower risk of severe COVID-19,^3^ the pandemic significantly impaired their lives.^4^ As these participants were assessed before the pandemic, we were able to evaluate the impact of the COVID-19 pandemic on their health and living conditions.

## What will be the new areas of research?

The 11- and 15-year visits prior to the pandemic, as well as the COVID-19 impact study at age 17, covered issues that were relevant to the age of the cohort, focusing on the following six groups of outcome variables: (i) mental health; (ii) body composition; (iii) risk factors for non-communicable diseases (NCDs); (iv) clinical conditions; (v) human capital; and (vi) violence and stress. In addition, the COVID-19 impact study collected information on the impact of the pandemic on participants and their mothers or guardians.

At both the 11- and 15-year follow-ups, the participants responded to face-to-face interviews without maternal assistance. They answered a general questionnaire, with questions about their life, day-to-day life, and health. Questions about sleep, physical activity, leisure and screen time were expanded compared with the 6-year follow-up. Questions about perception of the school environment, bullying suffered, stressful events, scale of face and body perception, and locus of control were included. The Food Frequency Questionnaire (FFQ) had the number of food items increased. At age 11, questions about diet to lose weight and coffee consumption were asked directly to the participant, without the assistance of mother, and the rest of the instrument was applied to mother. At age 15, questions were asked directly to the participant. The 15-year follow-up included questions about work, subjective social status, issues related to self-esteem and emotional control, dating, pregnancy and children, eating behaviour and headaches.

A confidential questionnaire, applied to the participant, was introduced at age 11. With the consent of mother or guardian, the participant answered a self-administered questionnaire, including questions about smoking, alcohol, fights, violence and relationship with parents. At 15 years of age, this questionnaire was expanded further, with questions about drug use, violence, victimization experiences, perpetrated bullying, sexual development and intra-family relationships. At 11 and 15 years of age, self-esteem, emotion regulation and locus of control (LoC) and executive functions were assessed. Stress response was evaluated using hair samples for cortisol quantification at 15 years and in the COVID-19 impact study. The COVID-19 impact study focused on the effect of the pandemic on the lives of young people and their families (Table 1).

**Table 1.** Main categories of variables collected in the most recent visits (11- and 15-year follow-ups and COVID-19 impact study) for adolescents and their mothers, Pelotas 2004 Birth Cohort Study

Main variables collected in each group	11-year follow-up	15-year follow-up	COVID-19 impact study
Mental health			
Development and Well-Being Assessment (DAWBA)	O	O	
Executive function (TEA-ch and CANTAB)	X	X	
Locus of control (LoC)	X	X	X
Self-esteem		X	X
Emotional control		X	X
Feelings and behaviours		X	X
Self-control		X	
Strengths and Difficulties Questionnaire (SDQ)	O	O	X
Wellbeing	X (face scale)	X (face scale)	
Inventory of Callous-Unemotional Traits (ICU)		X	
Body composition			
Anthropometric measurements	X	X	
Air-displacement plethysmography (Bod Pod^®^)	X	X	
Whole-body dual-energy X-ray absorptiometry (Lunar Prodigy, GE Healthcare^®^)	X	X	
Three-dimensional photonic scan (3D Photonic Scanner TC2^®^)	X		
Blood pressure	X	X	
Self-reported weight			X
Risk factors for NCDs			
Dietary evaluation (FFQ)	O-X	X	
Coffee intake	X	X	
Smoking	X	X	X
Alcohol intake	X	X	
Eating behaviours		X	
Physical activity	X	X	X
Sedentary behaviour		X	
Sexual behaviour		X	
Leisure and screen time	X	X	X
Sleep	X	X	X
Clinical conditions			
General health	O	O-X	O-X
Hearing and vision	O	O	
Pneumonia	O	O	
Urinary tract infection	O	O	
Medical appointments	O	O	
Hospitalizations	O	O	
Medications used	O	O	O
Oral health	O	O-X	
Use of oral health services	O	X	
Headaches		X	
Wheezing	O	X	
Accidents	O	X	
Menstruation (for girls)	X	X	
Body perception	X	X	
Human capital			
Socioeconomic status	O	O	O
Education	O-X	O-X	X
Employment		X	X
Reproductive history		X	
Mother’s socioeconomic status	O	O	O
Mother’s marital status	O	O	
Mother’s education	O	O	
Mother’s employment	O	O	O
Mother’s reproductive history	O	O	
Perception about justice and opinion about laws		X	
Society perception		X	
Rules at home		X	
Subjective socioeconomic position		X	X
Violence and stress			
Child maltreatment (CTSPC)	O	O	O
Perpetrated violence	X	X	X
Victimization	X	X	
Hair cortisol		X	X
Stress			X
Stressful events	X	X	X
Bullying	X	X	
COVID-19 pandemic			
COVID-19 diagnosis			O-X
Social distancing and measures of social distancing for COVID-19			O-X
Maternal general health, mental health and cognition			
Mother’s general health	O	O	O
Mother’s smoking	O	O	O
Mother’s alcohol intake		O	
Edinburgh Postnatal Depression Scale (EPDS)	O	O	O
IQ (WASI)		O	
Mini International Neuropsychiatric Interview (MINI)		O	
Quality of life	O		

Circles denote the variables that were collected in an interview with the mother, indicates that they were collected in an interview with the participants.

CANTAB, Cambridge Neuropsychological Test Automated Battery; CTSPC, Parent-Child Conflict Tactics Scales; FFQ, Food Frequency Questionnaire; IQ, intelligence quotient; NCDs, non-communicable diseases; WASI, Wechsler Abbreviated Scale of Intelligence.

Data on mothers’ general health, mental health and cognition were also collected at these follow-ups (Table 1).

## Who is in the cohort?

**Figure 1.**
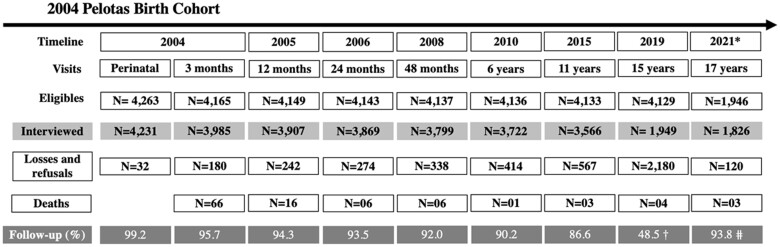
Description of the 2004 Pelotas Birth Cohort visits, assessed participants and follow-up rates. ^a^Follow-up interrupted due to the COVID-19 pandemic. ^b^COVID-19 assessment. ^c^The follow-up rate refers to the total number of participants evaluated in 2019

The new waves of data collection of the 2004 Pelotas birth cohort were carried out in 2015, 2019 and 2021, when the participants were aged 11, 15 and 17 years, respectively. Figure 1 shows the number of newborns enrolled in the cohort at baseline and in subsequent waves of data collection, as well as the number lost to follow-up and death.

### 11-year follow-up

By the time of the 11-year visit, 98 deaths had been identified among the 4231 original cohort members. All surviving cohort members (*n * = * *4133) were assessed in 2015 for the age-11 visit, and 3566 were interviewed face-to-face and completed the confidential questionnaire. Adding those known to have died, the follow-up rate was 86.6%.

Aiming to reduce losses to follow-up, several search strategies were adopted simultaneously, including tracing the participants at the elementary schools in the city of Pelotas and updating the registration data through phone calls using the contacts obtained at the 6-year visit. For those not found, a member of the study team visited the last known address of the family. Simultaneously, advertisements were placed in the city local media (newspaper and radio) informing people about the study and inviting participants to contact the study team.

The 11-year assessments were performed at the research clinic of the Epidemiologic Research Centre at Federal University of Pelotas. The average age of interviewees was 10.9 years (range, 10.1–11.7 years), and 51.5% of interviewed participants were boys. Follow-up rates according to baseline characteristics are presented in Table 2. Follow-up rates were highest among children of families in the intermediate income quintiles (*P *<0.001).

**Table 2.** Follow-up rates at the 11- and 15-year waves, according to baseline characteristics, Pelotas 2004 Birth Cohort Study (2015, 2019)

Characteristic	Original cohort (*n* =4231)	Follow-up rates			
		**11 years (*n* =3566)** ^a^	***P*** *	**15 years (*n* =1949)** ^b^	***P*** *
Age, mean (range)		10.9 (10.1–11.7)		15.7 (15.0–16.1)	
Maternal age at birth (years)	*n* = 4227 (missing, *n* = 4)		0.056		<0.001
<20	799 (18.9%)	693 (86.7%)		374 (46.8%)	
20–34	2865 (67.8%)	2462 (85.9%)		1359 (47.4%)	
≥35	563 (13.3%)	505 (89.7%)		317 (56.3%)	
Maternal education at birth (years)	*n* = 4186 (missing, *n* = 45)		0.123		0.242
0–4	654 (15.6%)	550 (84.1%)		302 (46.2%)	
5–8	1731 (41.4%)	1506 (87.0%)		833 (48.1%)	
≥9	1801 (43.0%)	1569 (87.1%)		898 (49.9%)	
Self-reported maternal skin colour	*n* = 4181 (missing, *n* = 50)		0.455		0.214
White	2581 (61.7%)	2243 (86.9%)		1270 (49.2%)	
Black, brown or other	1600 (38.3%)	1377 (86.1%)		755 (47.2%)	
Maternal smoking during pregnancy	*n* = 4229 (missing, *n* = 2)		0.086		0.270
No	3067 (72.5%)	2673 (87.2%)		1503 (49.0%)	
Yes	1162 (27.5%)	989 (85.1%)		547 (47.1%)	
Family income at birth (quintiles)	*n* = 4229 (missing, *n* = 2)		<0.001		0.002
1 (poorest)	872 (20.6%)	726 (83.3%)		396 (45.4%)	
2	854 (20.2%)	740 (86.7%)		408 (47.8%)	
3	816 (19.3%)	726 (89.0%)		427 (52.3%)	
4	857 (20.3%)	769 (89.7%)		447 (52.2%)	
5 (wealthiest)	830 (19.6%)	701 (84.5%)		372 (44.8%)	
Sex	*n* = 4231 (missing, *n* = 0)		0.528		0.580
Male	2195 (51.9%)	1893 (86.2%)		1055 (48.1%)	
Female	2036 (48.1%)	1770 (86.9%)		996 (48.9%)	
Gestational age (weeks)	*n* = 4215 (missing, *n* = 16)		0.405		0.141
<34	140 (3.3%)	121 (86.4%)		79 (56.4%)	
34–36	472 (11.2%)	418 (88.6%)		223 (47.3%)	
≥37	3603 (85.5%)	3110 (86.3%)		1737 (48.2%)	
Preterm birth	*n* = 4215 (missing, *n* = 16)		0.249		0.630
No	3603 (85.5%)	3110 (86.3%)		1737 (48.2%)	
Yes	612 (14.5%)	539 (88.1%)		302 (49.4%)	
Birthweight (grams)	*n* = 4226 (missing, *n* = 5)		0.248		0.178
<2500	423 (10.0%)	370 (87.5%)		223 (52.7%)	
2500–3499	2693 (63.7%)	2314 (85.9%)		1292 (48.0%)	
≥3500	1110 (26.3%)	975 (87.8%)		532 (47.9%)	
Month of birth	*n* = 4229 (missing, *n* = 2)		0.776		<0.001
January to March	1060 (25.1%)	925 (87.3%)		746 (70.4%)	
April to June	1108 (26.2%)	961 (86.7%)		620 (56.0%)	
July to September	1082 (25.6%)	937 (86.6%)		517 (47.8%)	
October to December	979 (23.1%)	839 (85.7%)		167 (17.1%)	

a The numerator used to calculate the follow-up rates was composed of 3566 assessed adolescents plus 98 deaths.

b The numerator used to calculate the follow-up rates was composed of 1949 assessed adolescents plus 102 deaths.

* Chi square test.

### 15-year follow-up

We aimed to interview all surviving cohort members in 2019 at age 15 years. Similar procedures to the 11-year follow-up were used. Furthermore additional strategies were used, including searches on social networks (Facebook and Instagram), contacting them through WhatsApp and encouraging participants to contact friends born in 2004, inviting them to visit the research clinic.

A total of 102 deaths were identified among the 4231 original cohort members until the 15-year follow-up. Of the 4129 remaining participants, 1949 were assessed at the research clinic before the COVID-19 pandemic. Added to those known to have died, this represents a 48.5% follow-up rate (Figure 1). For the traced participants, 1924 interviews were done with both the adolescent and the mother or guardian, two were done with the adolescent and partially with the mother or guardian, and 23 assessments were done with the mother or guardian and partially with the adolescent, mainly because those participants had intellectual and/or physical disability.

Follow-up rates were higher among adolescents of older mothers (≥35 years old; *P *<0.001), from families in the intermediate income quintile groups (*P * = * *0.002). The average age of the interviewees was 15.7 years (range, 15.0–16.1 years), and 51.4% of them were boys.

### COVID-19 impact study, age 17 years

The assessments for the COVID-19 impact study were performed in the participants’ homes from September to December 2021. Mother and adolescent answered several questionnaires about the adolescent’s behaviour and health, by trained interviewers. The participants assessed at 15-year were eligible for this study. A total of 1826 participants were interviewed. Added to the three deaths identified in the period, the follow-up rate was 93.8%. Out of the assessed participants, 1799 interviews were done with both adolescent and mother or guardian, five with only the adolescent, and 22 with only the mother, mainly due to the adolescent's intellectual disability diagnosis.

We traced participants based on addresses and contacts recorded during the 15-year visit. We invited mothers to participate through telephone calls, and scheduled a visit for the mothers and adolescents to receive the interviewer at their households. When contact by telephone was not possible, the interviewer went directly to the adolescent’s home to perform the interview or to schedule the visit for another day.

Follow-up rates were higher among adolescents from older mothers (≥35 years old) (*P* = 0.004), with higher education (≥9 years of schooling) (*P* = 0.002), who were non-smokers (*P* = 0.003) and from intermediate income quintiles (*P* = 0.019). The mean age at interview was 17.4 years (range, 16.7–18.0 years) and 51.9% were boys (Table 3).

**Table 3.** Follow-up rate for the COVID-19 impact study, according to baseline characteristics, Pelotas 2004 Birth Cohort Study, 2021

Characteristics	15 years (*n* = 1949)	**Follow-up rate, COVID-19 (*n* = 1826)** ^a^	***P*** *
Age, mean (range)		17.4 (16.7–18.0)	
Maternal age at birth (years)	*n* = 1949 (missing, *n* = 0)		0.004
<20	350 (18.0%)	312 (89.1%)	
20–34	1296 (66.5%)	1208 (93.2%)	
≥35	303 (15.5%)	290 (95.7%)	
Maternal education at birth (years)	*n* = 1935 (missing, *n* = 14)		0.002
0–4	277 (14.3%)	254 (91.7%)	
5–8	790 (40.8%)	717 (90.8%)	
≥9	868 (44.9%)	825 (95.1%)	
Self-reported maternal skin colour	*n* = 1925 (missing, *n* = 24)		0.921
White	1220 (63.4%)	1132 (92.8%)	
Black, brown or other	705 (36.6%)	655 (92.9%)	
Maternal smoking during pregnancy	*n* = 1949 (missing, *n* = 0)		0.003
No	1440 (73.9%)	1352 (93.9%)	
Yes	509 (26.1%)	458 (90.0%)	
Family income at birth (quintiles)	*n* = 1949 (missing, *n* = 0)		0.019
1 (poorest)	366 (18.8%)	332 (90.7%)	
2	383 (19.6%)	354 (92.4%)	
3	407 (20.9%)	371 (91.2%)	
4	431 (22.1%)	404 (93.7%)	
5 (wealthiest)	362 (18.6%)	349 (96.4%)	
Sex	*n* = 1949 (missing, *n* = 0)		0.161
Male	996 (51.1%)	917 (92.1%)	
Female	953 (48.9%)	893 (93.7%)	
Gestational age (weeks)	*n* = 1946 (missing, *n* = 3)		0.184
<34	45 (2.3%)	44 (97.8%)	
34–36	212 (10.9%)	192 (90.6%)	
≥37	1689 (86.8%)	1571 (93.0%)	
Preterm birth	*n* = 1946 (missing, *n* = 3)		0.492
No	1689 (86.8%)	1571 (93.0%)	
Yes	257 (13.2%)	236 (91.8%)	
Birthweight (grams)	*n* = 1948 (missing, *n* = 1)		0.501
<2500	173 (8.9%)	162 (93.6%)	
2500–3499	1254 (64.3%)	1169 (93.2%)	
≥3500	521 (26.8%)	478 (91.8%)	
Month of birth	*n* = 1949 (missing, *n* = 0)		0.430
January to March	722 (37.0%)	667 (92.4%)	
April to June	600 (30.8%)	560 (93.3%)	
July to September	483 (24.8%)	445 (92.1%)	
October to December	144 (7.4%)	138 (95.8%)	

a The numerator used to calculate the follow-up rates was composed of 1826 assessed adolescents plus three deaths.

* Chi square test.

## What has been measured?

The questionnaires included sections applied by trained interviewers to the mothers or adult caregivers and other parts to the adolescents. Table 1 summarizes the information collected from mothers and adolescents.

At 11 and 15 years of age, self-esteem, emotion regulation and locus of control (LoC) were assessed using the self-report version of the Rosenberg Self-esteem Scale, the Emotion Regulation Index for Children and Adolescents and the Nowick-Strickland Internal-External Scale, respectively. Executive functions were assessed using the Test of Everyday Attention for Children (TEA-ch) and the Cambridge Automated Neuropsychological Test Battery (CANTAB) at 11 and 15 years, respectively. Mental health was evaluated using the Development and Well-Being Assessment (DAWBA)^5^ and the Strengths and Difficulties Questionnaire (SDQ) instruments at both ages. To ascertain the presence and degree of the adolescent maltreatment, the Parent-Child Conflict Tactics Scales (CTSPC)^6^ were applied for the mothers in all follow-ups.

Anthropometric measurements including body weight, standing and sitting height and waist and abdominal circumferences were evaluated at 11 years of age. Abdominal and arm circumferences were taken with a non-extensible tape (CESCORF^®^). At 15-year follow-up, waist and hip circumferences were measured using a non-extensible tape (CARDIOMED^®^). Height measures were taken with a stadiometer (Harpenden^®^) (maximum 2.06-m and 1-mm precision). Because the COVID-19 impact study was conducted at the households, only self-reported weight and height were collected.

Body composition and shape were assessed by air-displacement plethysmography^7^ (Bod Pod^®^) and whole-body dual-energy X-ray absorptiometry^8^ (Lunar Prodigy, GE Healthcare^®^) at 11- and 15-year follow-ups. Body circumferences were measured using three-dimensional photonic scan^9^ (3D Photonic Scanner TC2^®^nometer model HEM 742) using the right arm with the adolescent seated after at least 5-min rest, at 11-year follow-up. Accelerometer devices for physical activity measurement were worn using the ActiGraph wGT3X-BT and wActiSleep-BT models at both 11- and 15-year follow-ups. The accelerometer was positioned for use on the wrist and required to stay for 7 full days. For the COVID-19 life impact study, a brief questionnaire about physical activity in leisure time was asked.

At the 11-year follow-up, the Pittsburgh Sleep Quality Index (PSQI) instrument was used to evaluate sleep duration and quality. At 15 years, besides the PSQI and questions about insomnia, the Munich ChronoType Questionnaire (MCTQ) and Epworth Sleepiness Scale were applied. During the reassessment in the COVID-19 life impact study, the MCTQ was used to evaluate sleep habit times, duration and social jetlag. Moreover, the movement data obtained by actigraphy at the 11- and 15-year follow-ups were used to estimate sleep parameters such as sleep duration and sleep efficiency.

A small portion of hair was collected from the posterior part of the head (the crown) by trained fieldworkers at the 15-year visit and in the COVID-19 life impact study. The hair was cut as close as possible to the scalp using chirurgical scissors. The hair collection aimed to assess the cortisol concentration in the 3 months before the interview, by measuring the cortisol concentration in the 3 cm closest to the scalp.

To estimate the impact of COVID-19 on the life of participants and their families, at the COVID-19 impact study, the information collected covered: access to monetary help by the government; financial situation during the pandemic; life habits; mask use; social distancing; COVID-19 diagnosis and death in participants, parents, other family members and friends; school and work during the pandemic; fear of getting contaminated with COVID-19; and subjective evaluation of COVID-19 pandemic impact on everyday life. Although the pandemic led to the 15-year assessment fieldwork being halted in early 2020, this provided a unique opportunity to assess the impact of COVID-19 as in a natural experiment.

The Edinburgh Postnatal Depression Scale (EPDS)^10^ was answered by mothers to evaluate depression symptoms in all follow-ups. At the 15 years of age, the mother’s mental health was also assessed for the first time through the Mini International Neuropsychiatric Interview (MINI). Mothers also had the Intelligence Quotient evaluated by means of the application of the Wechsler Abbreviated Intelligence Scale (WASI), in the same follow-up.

The data were entered directly by trained interviewers in RedCap (Research Electronic Data Capture).^11^ In case of confidential questionnaires, the participants entered the answers directly in the software, in a private room.

## What has it found? Key findings and publications

Several papers have been published using data from the 11- and 15-year follow-up visits. Analyses mainly involved children’s body composition,^12^ diet,^13^^,^^14^ sleep and mental health outcomes, ^15–26^ as well as general and mental health of their mothers. Table 4 presents several results from the 11- and 15-year visits.

**Table 4.** Comparison of selected results collected at the 11- and 15-year follow-up waves of the 2004 Pelotas Birth Cohort

Indicator	11 years		15 years	
	*n*	**% (95% CI** ^d^ **)**	*n*	**% (95% CI** ^d^ **)**
Any mental disorder^a^	499	14.0 (12.9–15.1)	284	14.6 (13.1–16.3)
Maternal depression screening^b^	974	27.4 (25.9–28.9)	545	31.3 (29.1–33.4)
Obesity	755	21.8 (20.4–23.2)	267	13.9 (12.4–15.5)
Ever smoked	49	1.4 (1.0–1.8)	383	20.2 (18.3–22.0)
Ever consumed alcohol	279	8.0 (7.1–8.9)	1471	78.4 (76.5–80.3)
Wellbeing^c^	1762	50.0 (48.3–51.6)	317	16.0 (14.4–17.6)
Attending school at the time of follow-up	3530	99.9 (99.8–99.9)	1949	97.9 (97.2–98.5)
Vision problem	622	17.5 (16.3–18.8)	528	27.7 (25.8–29.8)
Allergic rhinitis	857	24.1 (22.7–25.5)	537	28.2 (26.3–30.3)
Pneumonia	228	6.4 (5.6–7.2)	82	4.3 (3.5–5.3)
Involvement in fights	486	14.0 (12.9–15.2)	199	10.5 (9.2–12.0)

a SDQ: Strengths and Difficulties Questionnaire (score ≥17 pts).

b EPDS: Edinburgh Postnatal Depression Scale (score ≥10 pts).

c Very happy in the year before the follow-up (according to face scale).

d The 95% CI was derived from the central estimate observed at each visit.

The prevalence of adolescents with a high number of mental health problem symptoms (SDQ score ≥17) did not change from 11 to 15 years of age (14.0%; 95% CI 12.9–15.1%; and 14.6%; 95% CI 13.1–16.3%, respectively). Among mothers on the other hand, the prevalence of depressive symptoms, based on an EPDS score ≥10, increased from 27.4% (95% CI 25.9–28.9%) at the 11-year visit to 31.3% (95% CI 29.1–33.4%) at the 15-year visit.

The prevalence of adolescents’ obesity, using the body mass index-for-age >2 standard deviations by the World Health Organization (WHO) criterion,^27^ decreased from 21.8% (95% CI 20.4–23.2%) at 11 years to 13.9% (95% CI 12.4–15.5%) at 15 years.

Regarding alcoholic beverages and cigarette experimentation, prevalence increased considerably between age 11 and 15, as expected. At age 11, 1.4% (95% CI 1.0–1.8%) of participants reported having tried cigarettes and 8.0% (95% CI 7.1–8.9%) reported having tried alcoholic beverages. At age 15, these proportions were 20.2% (95% CI 18.3–22.0%) and 78.4% (95% CI 76.5–80.3%), respectively.

Self-reported involvement in fights during the year before the interview presented a reduction from 14.0% (95% CI 12.9–15.2%) at age 11 to 10.5% (95% CI 9.2–12.0%) at the 15-year follow-up.

There was a small decrease in the proportion of adolescents attending school in the year before the interview. Almost the total sample attended school at the 11-year follow-up (99.9%; 95% CI 99.8–99.9%), but at the 15-year visit, this dropped to 97.9% (95% CI 97.2–98.5%).

Results from the COVID-19 impact study showed the following.

- In all, 14.5% (95% CI: 13.0–16.2%) of the adolescents reported having had COVID-19 infection.
- In all, 3.4% (95% CI: 2.7–4.4%) reported having friend(s) who died due to COVID-19 infection.
- Regarding the social restrictions imposed by the local authorities at the beginning of the pandemic period, 26.4% (95% CI: 24.4–28.5%) of the participants did not leave the house, and 43.6% (95% CI: 41.3–45.9%) left the house only for essential activities and for work, whereas 6.8% (95% CI: 5.8–8.1%) reported leading a normal life as it was before the COVID-19 pandemic.
- In all, 13.9% (95% CI: 11.9–16.3%) were not studying by the time of the interview.
- In all, 30.0% (95% CI: 27.7–32.3%) had been anxious about food shortages at home during the pandemic.
- In all, 6.4% (95% CI: 5.3–7.7%) felt vulnerable as they did not have access to a mask and hygiene products.
- In all, 41.0% (95% CI: 38.6–43.5%) reported family financial problems during the pandemic.
- In all, 42.1% (95% CI: 39.7–44.6%) presented symptoms of insomnia.
- In all, 52.5% (95% CI: 50.1–54.9%) reported weight gain during the pandemic.
- In all, 59.5% (95% CI: 57.1–62.0%) reported having being hungrier, whereas 21.9% (95% CI: 19.9–24.0%) reported having been less hungry during the pandemic.

## What are the main strengths and weaknesses?

One of the main strengths of the Pelotas 2004 cohort is its population-based design, with very high follow-up rates compared with other study sites. Another strength is the potential for comparative analyses with the other three Pelotas birth cohort studies started in 1982, 1993 and 2015, providing a unique opportunity to assess changes in health exposures and outcomes over decades in the same city.^28^

The premature interruption of the 15-year follow-up due to the COVID-19 pandemic led to a reduction in the retention rate, which can potentially limit the statistical power for some association analyses and potentially increase the selection bias. The main limitation of this study is that the follow-up rate of 48.5% at 15 years is far below the 86.6% follow-up rate at 11 years. However, for many maternal and child characteristics, the sample was comparable to the original cohort. The only two variables that showed an imbalance at the 15-year follow-up were family income and maternal age at birth, in which children from older mothers and intermediate income quintiles at birth were over-represented. As shown in previous analyses, children of mothers from both low and high socioeconomic position have also been lost to follow-up in the other Pelotas cohort studies.^1^^,^^2^^,^^29–31^ It is worth highlighting several difficulties in carrying out fieldwork via home visits during the COVID-19 pandemic. Issues regarding safety protocol for the interviewers, such as training and supervising the use of individual protection equipment (IPE), and adhering to recommended social distancing measures, were challenges to overcome when trying to balance the interviewer’s and participant’s safety and privacy for the participant’s responses. Another problem faced during the fieldwork was the high number of cancelled linterviews because the participant or any member of his/her family had COVID-19 or because he/she was afraid of COVID-19 contamination. Several interviews had to be rescheduled, and a high number of interviewers had to be removed from the activities due to COVID-19 infection, making it necessary to carry out multiple instances of fieldworker training over the study period.

## Can I get hold of the data? Where can I find out more?

We have successfully collaborated with investigators from many countries around the world. We recently established partnerships with researchers from academic institutions in the USA, Canada, Mexico, Chile, Colombia, the UK, Norway, Portugal, Spain and South Africa. We also collaborate with Brazilian institutions and participate in the Brazilian Ribeirão Preto-Pelotas-São Luiz (RPS) birth cohorts consortium. Exchange of doctoral or postdoctoral fellows between other institutions and Pelotas is very welcome. For further information on postgraduate training, see the programme website at [http://epidemio-ufpel.org.br/site/content/coorte_2004/index.php] or e-mail the investigators involved in the research areas of interest. The questionnaires and interviewer guides from all follow-up visits are available in electronic formats at [http://www.epidemio-ufpel.org.br/site/content/coorte_2004/questionarios.php]. We welcome collaborative research proposals that will include local and external scientists, particularly involving our local postgraduate students. For any queries about collaboration, please contact the corresponding author.

## Ethics approval

In all phases of the study, ethics approval was obtained from the Medical School Ethics Committee of the Federal University of Pelotas and the University of São Paulo, and full informed consent was provided by parents or legal guardians. Written informed consent was also provided by the adolescents. The approval protocol numbers provided by Medical School Ethics Committee of the Federal University of Pelotas were 889.753 (11-years), 3.554.667 (15-years) and 4.878.509 (COVID-19 impact study). The Medical School Ethics Committee of the University of São Paulo also approved the COVID-19 impact study under protocol number 4.951.457.

## Data Availability

The data underlying this article will be shared on reasonable request to the corresponding author.

## References

[1] Santos IS , Barros AJD, Matijasevich A, Domingues MR, Barros FC, Victora CG. Cohort profile: the 2004 Pelotas (Brazil) birth cohort study. Int J Epidemiol 2011;40:1461–68. 20702597 10.1093/ije/dyq130 PMC3235016

[2] Santos IS , Barros AJD, Matijasevich A et al Cohort profile update: 2004 Pelotas (Brazil) Birth Cohort Study. Body composition, mental health and genetic assessment at the 6 years follow-up. Int J Epidemiol 2014;43:1437. 25063002 10.1093/ije/dyu144 PMC4190519

[3] Bhopal SS , Bagaria J, Olabi B, Bhopal R. Children and young people remain at low risk of COVID-19 mortality. Lancet Child Adolesc Health 2021;5:e12–13. 33713603 10.1016/S2352-4642(21)00066-3 PMC7946566

[4] Cost KT , Crosbie J, Anagnostou E et al Mostly worse, occasionally better: impact of COVID-19 pandemic on the mental health of Canadian children and adolescents. Eur Child Adolesc Psychiatry 2022;31:671–84. 33638005 10.1007/s00787-021-01744-3 PMC7909377

[5] Fleitlich-Bilyk B , Goodman R. Prevalence of child and adolescent psychiatric disorders in southeast Brazil. J Am Acad Child Adolesc Psychiatry 2004;43:727–34. 15167089 10.1097/01.chi.0000120021.14101.ca

[6] Reichenheim M , Moraes C. Portuguese-language cross-cultural adaptation of the Parent-Child Conflict Tactics Scales (CTSPC), an instrument used to identify parental violence against children. Cad Saude Publica 2003;19:1701–12. 14999336 10.1590/s0102-311x2003000600014

[7] Fields D , Goran M, McCrory M. Body-composition assessment via air-displacement plethysmography in adults and children: a review. Am J Clin Nutr 2002;75:453–67. 11864850 10.1093/ajcn/75.3.453

[8] Kelly T , Wilson K, Heymsfield S. Dual energy X-ray absorptiometry body composition reference values from NHANES. PLoS One 2009;4:e7038. 19753111 10.1371/journal.pone.0007038 PMC2737140

[9] Wells J , Douros I, Fuller N, Elia M, Dekker L. Assessment of body volume using three-dimensional photonic scanning. Ann N Y Acad Sci 2000;904:247–54. 10865749 10.1111/j.1749-6632.2000.tb06460.x

[10] Santos IS , Matijasevich A, Tavares BF et al Validation of the Edinburgh Postnatal Depression Scale (EPDS) in a sample of mothers from the 2004 Pelotas Birth Cohort Study. Cad Saude Publica 2007;23:2577–88. 17952250 10.1590/s0102-311x2007001100005

[11] Harris PA , Taylor R, Thielke R, Payne J, Gonzalez N, Conde JG. Research Electronic Data Capture (REDCap) - a metadata-driven methodology and workflow process for providing translational research informatics support. J Biomed Inform 2009;42:377–81. 18929686 10.1016/j.jbi.2008.08.010 PMC2700030

[12] Santos LP , Santos IS, Matijasevich A, Barros AJD. Changes in overall and regional body fatness from childhood to early adolescence. Sci Rep 2019;9:1888. 30760792 10.1038/s41598-019-38486-x PMC6374425

[13] Costa CS , Assunção MCFA, Loret de Mola C et al Role of ultra-processed food in fat mass index between 6 and 11 years of age: a cohort study. Int J Epidemiol 2021;50:256–65. 32888008 10.1093/ije/dyaa141 PMC7938497

[14] Costa CS , Assunção MCFA, Vaz JS et al Consumption of ultra-processed foods at 11, 22 and 30 years at the 2004, 1993 and 1982 Pelotas Birth Cohorts. Public Health Nutr 2021;24:299–308. 32204744 10.1017/S1368980019004245 PMC10195471

[15] Bozzini AB , Maruyama JM, Munhoz TN et al Trajectories of maternal depressive symptoms and offspring's risk behavior in early adolescence: data from the 2004 Pelotas Birth Cohort Study. BMC Psychiatry 2021;21:18. 33413253 10.1186/s12888-020-03026-9 PMC7792177

[16] Bozzini AB , Rudi R, Munhoz TN, Santos IS, Barros AJD, Matijasevich A. Prevalence and early life factors associated with risk behaviors in adolescence: a population-based cohort study. J Adolesc Health 2019;64:S72–73.

[17] Carpena MX , Matijasevich A, Loret de Mola C, Santos IS, Munhoz TN, Tovo-Rodrigues L. The effects of persistent sleep disturbances during early childhood over adolescent ADHD, and the mediating effect of attention-related executive functions: Data from the 2004 Pelotas Birth Cohort. J Affect Disord 2022;296:175–82. 34607058 10.1016/j.jad.2021.09.053

[18] Carpena MX , Munhoz TN, Xavier MO et al The role of sleep duration and sleep problems during childhood in the development of ADHD in adolescence: findings from a population-based birth cohort. J Atten Disord 2020;24:590–600. 31617436 10.1177/1087054719879500

[19] Del-Ponte B , Anselmi L, Assunção MCFA et al Sugar consumption and attention-deficit/hyperactivity disorder (ADHD): a birth cohort study. J Affect Disord 2019;243:290–96. 30257225 10.1016/j.jad.2018.09.051 PMC6193136

[20] Maruyama JM , Pastor-Valero M, Santos IS, Munhoz TN, Barros FC, Matijasevich A. Impact of maternal depression trajectories on offspring socioemotional competences at age 11: 2004 Pelotas Birth Cohort. J Affect Disord 2019;253:8–17. 31009846 10.1016/j.jad.2019.03.072 PMC6609923

[21] Maruyama JM , Santos IS, Munhoz TN, Matijasevich A. Maternal depression trajectories and offspring positive attributes and social aptitudes at early adolescence: 2004 Pelotas Birth Cohort. Eur Child Adolesc Psychiatry 2021;30:1939–48. 33098444 10.1007/s00787-020-01665-7

[22] Matijasevich A , Pearson RM, Loret de Mola C et al Early child stimulation and attention-related executive functions at 11 years: 2004 Pelotas Birth Cohort Study. Eur Child Adolesc Psychiatry 2020;29:1265–76. 31748986 10.1007/s00787-019-01440-3

[23] Maruyama JM , Valente JY, Tovo-Rodrigues L et al Maternal depression trajectories in childhood, subsequent maltreatment, and adolescent emotion regulation and self-esteem: the 2004 Pelotas Birth Cohort. Eur Child Adolesc Psychiatry 2023;32:1935–45. 35731302 10.1007/s00787-022-02022-6 PMC9214189

[24] Bozzini AB , Maruyama JM, Santos IS et al Prevalence of adolescent's risk behaviors at 11 and 15 years of age: data from the 2004 Pelotas Birth Cohort. Braz J Psychiatry 2023;45:93–101. 10.47626/1516-4446-2022-2753 PMC10154011 36318481

[25] Munhoz TN , Santos IS, Barros AJD, Anselmi L, Barros FC, Matijasevich A. Perinatal and postnatal risk factors for disruptive mood dysregulation disorder at age 11: 2004 Pelotas Birth Cohort Study. J Affect Disord 2017;215:263–68. 28347949 10.1016/j.jad.2017.03.040 PMC5408904

[26] Martins-Silva T , Bauer A, Matijasevich A et al Educational performance and conduct problem trajectories from childhood to adolescence: observational and genetic associations in a Brazilian Birth Cohort. JCPP Adv 2022;2:e12105. 37431415 10.1002/jcv2.12105 PMC10242956

[27] de Onis M. WHO Child Growth Standards based on length/height, weight and age. Acta Paediatr Suppl 2007;95:76–85. 10.1111/j.1651-2227.2006.tb02378.x 16817681

[28] Barros FC , Victora CG. Commentary: A tale of many cities in one: the Pelotas (Brazil) Birth Cohorts, 1982–2015. Int J Epidemiol 2019;48:i89–93. 30883658 10.1093/ije/dyy214 PMC6422057

[29] Victora CG , Hallal PC, Araújo CLP, Menezes AMB, Wells JCK, Barros FC. Cohort Profile: The 1993 Pelotas (Brazil) Birth Cohort Study. Int J Epidemiol 2008;37:704–709. 17846051 10.1093/ije/dym177

[30] Gonçalves H , Assunção MCFA, Wehrmeister FC et al Cohort Profile update: The 1993 Pelotas (Brazil) Birth Cohort follow-up visits in adolescence. Int J Epidemiol 2014;43:1082–88. 24729426 10.1093/ije/dyu077 PMC4121560

[31] Gonçalves H , Wehrmeister FC, Assunção MCFA et al Cohort Profile Update: The 1993 Pelotas (Brazil) Birth Cohort follow-up at 22 years. Int J Epidemiol 2017;1–7. 29240909 10.1093/ije/dyx249 PMC6208268

